# Vitamin D—Effects on Skeletal and Extraskeletal Health and the Need for Supplementation

**DOI:** 10.3390/nu5010111

**Published:** 2013-01-10

**Authors:** Matthias Wacker, Michael F. Holick

**Affiliations:** Vitamin D, Skin and Bone Research Laboratory, Section of Endocrinology, Nutrition, and Diabetes, Department of Medicine, Boston University Medical Center, 85 East Newton Street, M-1013, Boston, MA 02118, USA; E-Mail: mwacker@bu.edu

**Keywords:** vitamin D, 25-hydroxyvitamin D, vitamin D deficiency, osteoporosis, fractures, cancer, type 2 diabetes mellitus, cardiovascular diseases, autoimmune diseases, infectious diseases

## Abstract

Vitamin D, the sunshine vitamin, has received a lot of attention recently as a result of a meteoric rise in the number of publications showing that vitamin D plays a crucial role in a plethora of physiological functions and associating vitamin D deficiency with many acute and chronic illnesses including disorders of calcium metabolism, autoimmune diseases, some cancers, type 2 diabetes mellitus, cardiovascular disease and infectious diseases. Vitamin D deficiency is now recognized as a global pandemic. The major cause for vitamin D deficiency is the lack of appreciation that sun exposure has been and continues to be the major source of vitamin D for children and adults of all ages. Vitamin D plays a crucial role in the development and maintenance of a healthy skeleton throughout life. There remains some controversy regarding what blood level of 25-hydroxyvitamin D should be attained for both bone health and reducing risk for vitamin D deficiency associated acute and chronic diseases and how much vitamin D should be supplemented.

## 1. Introduction

Vitamin D has been produced by phytoplankton for more than 500 million years [1] and is thought to be the oldest of all hormones whose function initially could have been the protection of ultraviolet-sensitive macromolecules including proteins, DNA and RNA, when these early forms of life were exposed to sunlight for photosynthesis. Later, after the evolution of ocean dwelling animals with vertebral skeletons ventured onto land, the maintenance of calcium homeostasis was a major physiological problem (as opposed to living in the calcium-rich ocean). It was vitamin D that ensured the efficient intestinal calcium absorption from dietary sources and ultimately was essential for the development and maintenance of a calcified mammalian skeleton [2]. Obtaining vitamin D from either sunlight or diet is still critical for most vertebrates for their skeletal health [1,3,4,5]. Over time, vitamin D has evolved into a hormone having numerous extraskeletal effects by regulating up to estimated 2000 genes [6,7].

Ethnical and gender differences in skin pigmentation indicate the evolutionary importance of a sufficient vitamin D supply. The varying degrees of depigmentation that evolved in order to permit UVB-induced synthesis of previtamin D_3_ when hominids migrated outside the tropics can be considered as a compromise solution to the conflicting physiological requirements of vitamin D synthesis and photoprotection that differ depending on latitude and thus warrant different degrees of skin pigmentation. An evolutionary selection pressure towards a lighter skin coloration going along with a higher ability to produce vitamin D seems not only to be exerted by living in geographic regions with a lower UV intensity but also by being female. Gender differences in skin pigmentation with females being lighter skinned than males in all populations for which data about the skin reflectance was available could be explained by the higher needs of vitamin D during pregnancy and lactation [8].

## 2. Vitamin D—Sources

The main sources of vitamin D are sunlight, supplements and diet [7] (Table 1).

**Table 1:** Sources of vitamin D_2_ and vitamin D_3_ [7]. Note: This table is modified and reproduced with permission from [7], Copyright © 2007 Massachusetts Medical Society.

Source	Vitamin D Content	
	IU = 25 ng	
	Chemical structures of vitamin D_2_ [9] and vitamin D_3_ [10]_._	
	Vitamin D_2_ (Ergocalciferol)	Vitamin D_3_ (Cholecalciferol)
	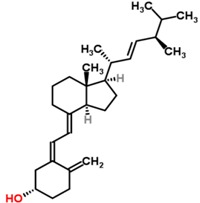	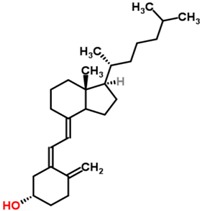
*Natural sources*		
Cod liver oil	~400–1000 IU/tsp vitamin D_3_	
Egg yolk	~20 IU/yolk vitamin D_3_ or D_2_	
Mackerel, canned	~250 IU/3.5 oz vitamin D_3_	
Salmon, canned	~300–600 IU/3.5 oz vitamin D_3_	
Salmon, fresh farmed	~100–250 IU/3.5 oz vitamin D_3_, vitamin D_2_	
Sardines, canned	~300 IU/3.5 oz vitamin D_3_	
Shiitake mushrooms, fresh	~100 IU/3.5 oz vitamin D_2_	
Shiitake mushrooms, sun dried	~1600 IU/3.5 oz vitamin D_2_	
Sunlight/UVB radiation	~20,000 IU equivalent to exposure to 1 minimal erythemal dose (MED) in a bathing suit. Thus, exposure of arms and legs to 0.5 MED is equivalent to ingesting ~3000 IU vitamin D_3_	
Tuna, canned	236 IU/3.5 oz vitamin D_3_	
*Fortified foods*		
Fortified breakfast cereals	~100 IU/serving usually vitamin D_3_	
Fortified butter	56 IU/3.5 oz usually vitamin D_3_	
Fortified cheeses	100 IU/3 oz usually vitamin D_3_	
Fortified margarine	429/3.5 oz usually vitamin D_3_	
Fortified milk	100 IU/8 oz usually vitamin D_3_	
Fortified orange juice	100 IU/8 oz vitamin D_3_	
Fortified yogurts	100 IU/8 oz usually vitamin D_3_	
Infant formulas	100 IU/8 oz vitamin D_3_	
*Pharmaceutical Sources in the United States*		
Drisdol (vitamin D_2_) liquid	8000 IU/mL	
Vitamin D_2_ (Ergocalciferol)	50,000 IU/capsule	
*Supplemental Sources*		
Multivitamin	400, 500, and 1000 IU vitamin D_3_ or vitamin D_2_	
Vitamin D_3_	400, 800, 1000, 2000, 5000, 10,000, 14,000, and 50,000 IU	

**Figure 1:**
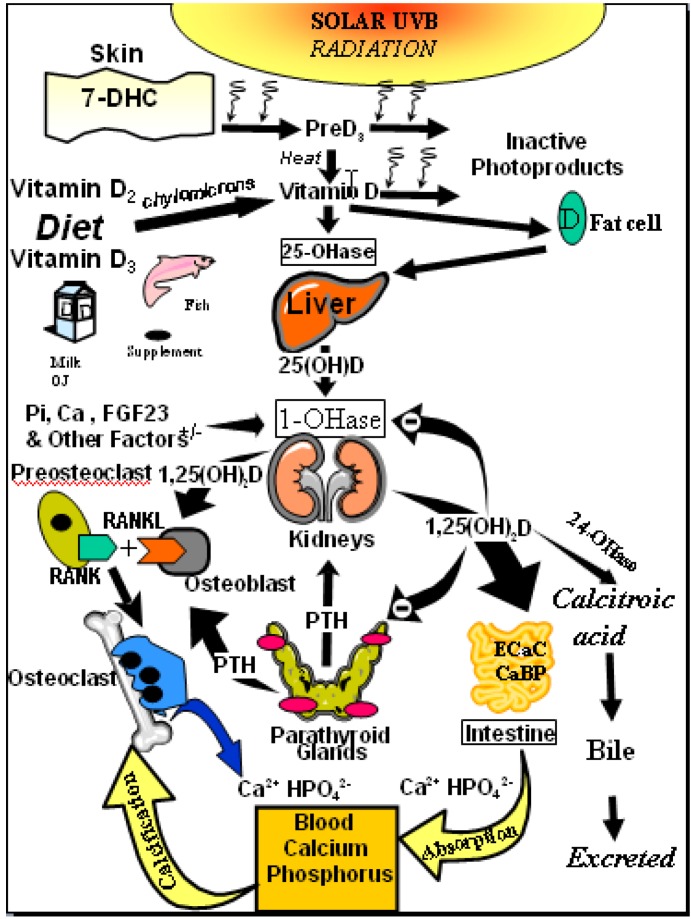
Schematic representation of the synthesis and metabolism of vitamin D for regulating calcium, phosphorus and bone metabolism [7]. During exposure to sunlight, 7-dehydrocholesterol in the skin is converted to previtamin D_3_. Previtamin D_3 _immediately converts by a heat dependent process to vitamin D_3_ [7,11,12]. Excessive exposure to sunlight degrades previtamin D_3_ and vitamin D_3_ into inactive photoproducts [13]. Vitamin D_2_ and vitamin D_3_ from dietary sources is incorporated into chylomicrons, transported by the lymphatic system into the venous circulation [14]. Vitamin D (D represents D_2_ or D_3_) made in the skin or ingested in the diet can be stored in and then released from fat cells. Vitamin D in the circulation is bound to the vitamin D binding protein which transports it to the liver where vitamin D is converted by the vitamin D-25-hydroxylase to 25-hydroxyvitamin D [25(OH)D]. This is the major circulating form of vitamin D that is used by clinicians to measure vitamin D status [7,15] (although most reference laboratories report the normal range to be 20–100 ng/mL, the preferred healthful range is 30–60 ng/mL) [7]. It is biologically inactive and must be converted in the kidneys by the 25-hydroxyvitamin D-1α-hydroxylase (1-OHase) to its biologically active form 1,25-dihydroxyvitamin D [1,25(OH)_2_D] [7,15,16,17]. Serum phosphorus, calcium, fibroblast growth factors (FGF-23) and other factors can either increase (+) or decrease (−) the renal production of 1,25(OH)_2_D [7]. 1,25(OH)_2_D feedback regulates its own synthesis and decreases the synthesis and secretion of parathyroid hormone (PTH) in the parathyroid glands [6,7]. 1,25(OH)_2_D increases the expression of the 25-hydroxyvitamin D-24-hydroxylase (24-OHase) to catabolize 1,25(OH)_2_D to the water soluble biologically inactive calcitroic acid which is excreted in the bile [7,18]. 1,25(OH)_2_D enhances intestinal calcium absorption in the small intestine by stimulating the expression of the epithelial calcium channel (ECaC) and the calbindin 9K (calcium binding protein; CaBP) [7,19,20]. 1,25(OH)_2_D is recognized by its receptor in osteoblasts causing an increase in the expression of receptor activator of NFκB ligand (RANKL). Its receptor RANK on the preosteoclast binds RANKL which induces the preosteoclast to become a mature osteoclast. The mature osteoclast removes calcium and phosphorus from the bone to maintain blood calcium and phosphorus levels [7,17]. Adequate calcium and phosphorus levels promote the mineralization of the skeleton [7]. Note: This figure is reproduced with permission from [21], Copyright © 2007 Michael F. Holick.

Exposure of human skin to solar UVB radiation (wavelengths: 290–315 nm) leads to the conversion of 7-dehydrocholesterol to previtamin D_3_ in the skin. Previtamin D_3_ is then rapidly converted to vitamin D_3_ (cholecalciferol) by temperature- and membrane-dependent processes [7,11,12] (Figure 1).

**Figure 2:**
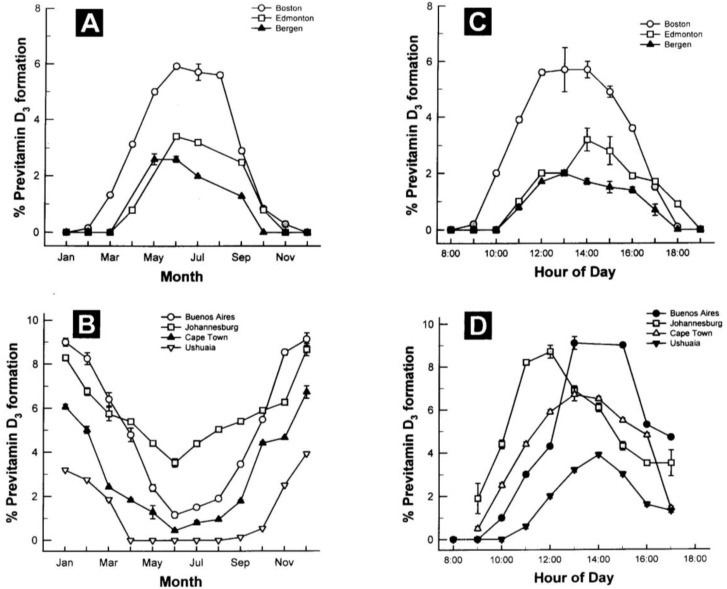
Influence of season, time of day, and latitude on the synthesis of previtamin D_3_ in Northern (**A** and **C**) and Southern hemispheres (**B** and **D**). The hour indicated in **C** and **D** is the end of the 1-h exposure time. Note: This figure is reproduced with permission from [13], Copyright © 2010 Humana Press.

The amount of vitamin D production in the skin depends on the incident angle of the sun and thus on latitude, season and time of the day. It is highest when the sun is in the zenith and a flattening of the incident angle leads to a reduced vitamin D production [17]. Whole body exposure to sunlight with one minimal erythema dose (MED), *i.e.*, the minimal dose leading to pink coloration of the skin 24 h after exposure, leads to vitamin D levels comparable to oral intake of 10,000 to up to 25,000 IU vitamin D_2_[16,22]. However, sun exposure during most of the winter at latitudes above and below ~33 degrees North and South, respectively, doesn’t lead to any production of vitamin D_3_ in the skin [16,23] (Figure 2). Other factors influencing the cutaneous vitamin D production adversely are an increase in skin pigmentation, aging, especially age >65 years and the topical application of a sunscreen [17].

The number of foods naturally containing vitamin D in significant amounts is very limited. Among these are oily fish such as salmon, sardines and tuna, and oils of the liver of some fish such as cod as well as sun-exposed mushrooms [7] (Table 1). To increase the content of vitamin D_2_ in mushrooms producers are irradiating them with UV radiation [24,25].

In the 1930s, the fortification of milk, sodas, bread and even beer became popular [26]; however, after several cases of presumed vitamin D intoxication in infants in the 1950s in Great Britain [27] strict regulations limiting vitamin D fortification to only margarine were introduced in Europe [14,28]. Due to a relatively high prevalence of lactose intolerance leading to an avoidance of milk by many adults, the fortification of orange juice in the US was introduced as a novel approach of enhancing the vitamin D status of the public in the 2003 and proved to be as effective as oral supplementation [26,29]. Other fortified foods include margarine, yogurt, infant formula, butter, cheese and breakfast cereals [7] (Table 1).

Vitamin D_2_ and vitamin D_3_ are available as oral over-the-counter supplements. In the US, only vitamin D_2_ is available as prescription drug [7,17]. Although there has been debate as to whether vitamin D_2_ is as effective as vitamin D_3_ in maintaining vitamin D status [30,31,32,33,34,35,36], other studies in children and adults have demonstrated that they are equally effective [29,37,38,39,40].

## 3. Vitamin D—Metabolism

Vitamin D from cutaneous synthesis or dietary/supplemental intake, is transported to the fat where it can be stored or to the liver for the first step of activation, the hydroxylation to 25-hydroxyvitamin D [25(OH)D], which is the major circulating form of vitamin D [7,15] and measured to assess a patient’s vitamin D status [7,16,41,42] (Figure 1).

25(OH)D is metabolized in the kidneys by the mitochondrial enzyme 25-hydroxyvitamin D-1α-hydroxylase (CYP27B1) to generate the systemically circulating active form, 1,25-dihydroxyvitamin D [1,25(OH)_2_D] [7,15,16,17]. The renal synthesis of 1,25(OH)_2_D is regulated by several factors including serum phosphorus, calcium, fibroblast growth factor 23 (FGF-23), parathormone (PTH) and itself [7]. CYP27B1 is also expressed extrarenally in a multitude of tissues [17,43], including bone, placenta, prostate, keratinocytes, macrophages, T-lymphocytes, dendritic cells, several cancer cells [44], and the parathyroid gland [45] and enables the production of 1,25(OH)_2_D. This active form of vitamin D is locally active and exerts auto- or paracrine effects [15,17].

1,25(OH)_2_D induces its own destruction by rapidly inducing the 25-hydroxyvitamin D-24-hydroxylase (CYP24A1), which leads to the multistep catabolism of both 25(OH)D and 1,25(OH)_2_D into biologically inactive, water-soluble metabolites including calcitroic acid [7,18] (Figure 1).

## 4. Vitamin D Receptor (VDR)—Distribution and Function

1,25(OH)_2_D, either produced in the kidneys [7] or extrarenally in the target tissues [15,17], is the ligand of the vitamin D receptor (VDR) whose widespread distribution across many tissues explains the myriad of physiological actions of vitamin D. By interacting with the VDR, a transcription factor [17,46], 1,25(OH)_2_D regulates directly and indirectly the expression of up to 2000 genes [6,7], many of whose promoters contain specific vitamin D response elements (VDRE). The VDR partners with other transcription factors, most importantly the retinoid X receptor (RXR) [47], and coactivators and corepressors provide target gene specificity [48,49,50]. A membrane-bound VDR may also exist and mediate more immediate, non-genomic actions of 1,25(OH)_2_D [44,51,52].

## 5. Prevalence of Vitamin D Deficiency and Insufficiency

25(OH)D is the vitamin D metabolite that is measured to assess a patient’s vitamin D status [7,17]. Vitamin D deficiency is diagnosed when 25(OH)D <20 ng/mL [16,53], vitamin D insufficiency is defined as 25(OH)D of 21–29 ng/mL, and 25(OH)D >30 ng/mL is considered sufficient, with 40–60 ng/mL being the preferred range [16]. Vitamin D intoxication usually doesn’t occur until 25(OH)D >150 ng/mL [7,16,23].

These reference values are in part based on the finding, that the decline of parathyroid hormone (PTH) concentrations with increasing 25(OH)D levels in adults reached its nadir asymptotically at a 25(OH)D of ~30–40 ng/mL in several studies [7,16,23,54,55,56]. However, a recent cross-sectional analysis of more than 300,000 paired serum PTH and 25(OH)D levels revealed no threshold, even at 25(OH)D levels >60 ng/mL, above which a further increase of the 25(OH)D level failed to further suppress PTH levels. The analysis also showed a strong age-dependency of the PTH-25(OH)D relationship [57].

According to studies in Canada, 30%–50% of children and adults are vitamin D deficient [58,59,60]. The National Health and Nutrition Examination Surveys 2001–2006 showed a prevalence of vitamin D deficiency of 33% [60,61]. Studies in Indian school children revealed a prevalence of severe vitamin D deficiency (<9 ng/mL) in more than 35% [62] and over 80% of pregnant women in India had 25(OH)D levels <22.5 ng/mL [63]. Also reports from Africa [64], Australia [65], Brazil [66], Middle East [67,68], Mongolia [69], and New Zealand [70] documented a high risk for vitamin D deficiency in both adults and children [60,71].

**Figure 3:**
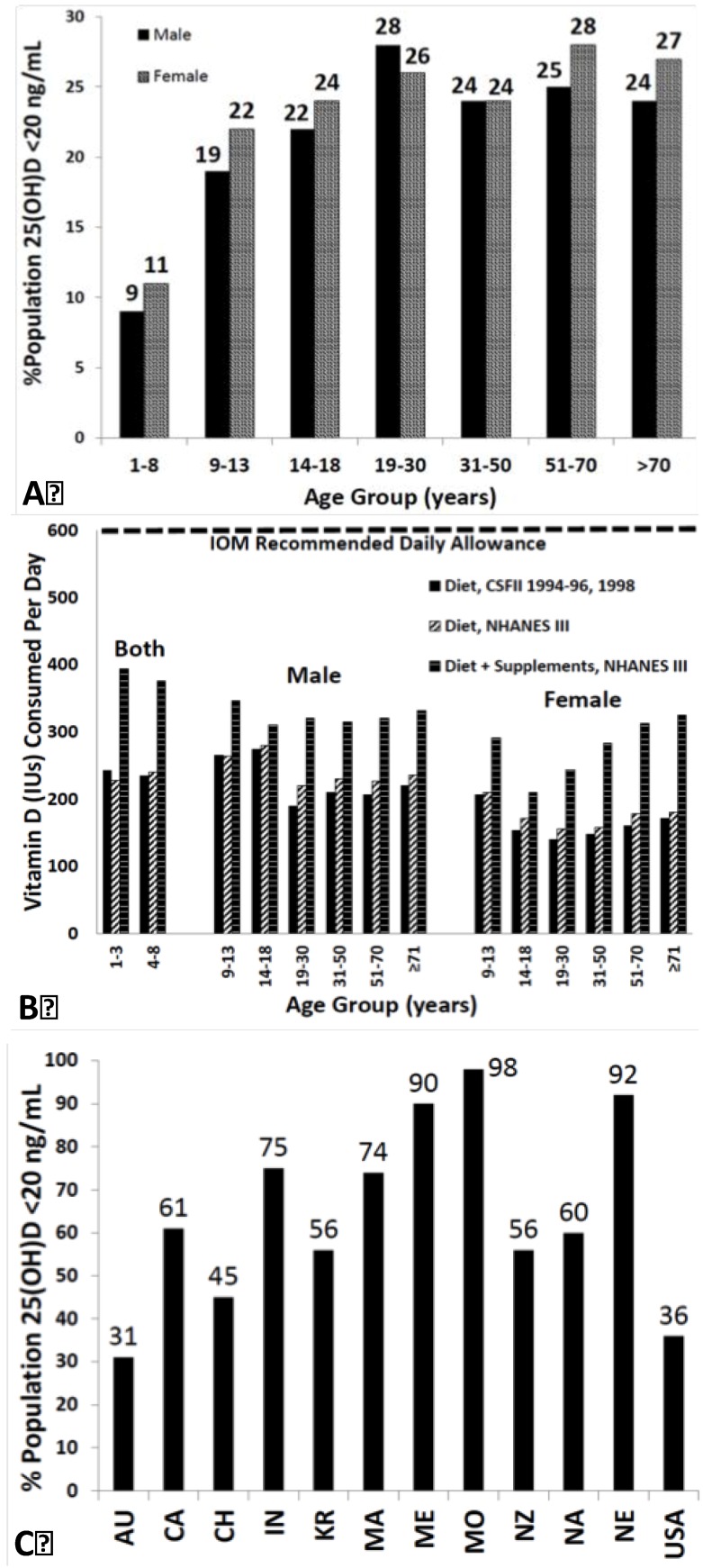
(**A**) Prevalence at risk of vitamin D deficiency defined as a 25-hydroxyvitamin D <12–20 ng/mL by age and sex: United States, 2001–2006. (**B**) Mean intake of vitamin D (IU) from food and food plus dietary supplements from Continuing Survey of Food Intakes by Individuals (CSFII) 1994–1996, 1998 and the Third National Health and Nutrition Examination Survey (NHANES III) 1988–1994. (**C**) Reported incidence of vitamin D deficiency defined as a 25-hydroxyvitamin D <20 ng/mL around the globe including Australia (AU), Canada (CA), China (CH), India (IN), Korea (KR), Malaysia (MA), Middle East (ME), Mongolia (MO), New Zealand (NZ), North Africa (NA), Northern Europe (NE), United States (USA) [60]. Note: This figure is reproduced with permission from [60], Copyright © 2012 The Endocrine Society.

Based on these findings, it has been estimated that 1 billion people worldwide are vitamin D deficient or insufficient [7,60] (Figure 3A–C).

## 6. Vitamin D and Calcium and Phosphorus Metabolism

Vitamin D plays an important role in the calcium and phosphorus metabolism and helps ensure adequate levels of these minerals for metabolic functions and bone mineralization [7]. 1,25(OH)_2_D increases the efficiency of intestinal calcium absorption from 10%–15% to 30%–40% by interacting with the VDR-RXR and thereby promoting the expression of an epithelial calcium channel and a calcium-binding protein [7,19,20]. Based on several experiments conducted in rodents [72,73] it has been estimated that 1,25(OH)_2_D also increases the intestinal phosphorus absorption from 50%–60% to approximately 80% [7,14].

Vitamin D also mediates indirect effects on calcium and phosphorus by regulating the PTH levels. The parathyroid glands have CYP27B1 activity and the local production of 1,25(OH)_2_D using 25(OH)D as substrate could inhibit the synthesis of PTH [74]. However, 25(OH)D could also directly suppress PTH synthesis by directly activating the VDR [75]. Vitamin D deficiency is associated with lower levels of serum-ionized calcium, a stimulus leading to increased PTH levels. Conversely, higher calcium levels that are associated with higher 25(OH)D levels, suppress the PTH secretion. PTH increases tubular calcium and decreases renal phosphorus reabsorption [14] (Figure 1). PTH also stimulates the production of 1,25(OH)_2_D with the above mentioned effects on calcium and phosphorus homeostasis [7,14]. Moreover, both PTH and 1,25(OH)_2_D stimulate osteoblasts to mobilize skeletal calcium stores [7,17] (Figure 1). Vitamin D deficiency leads to secondary hyperparathyroidism with PTH-enhanced 1,25(OH)_2_D production and is often associated with normal to high 1,25(OH)_2_D levels [7].

## 7. Bone Health

**Figure 4:**
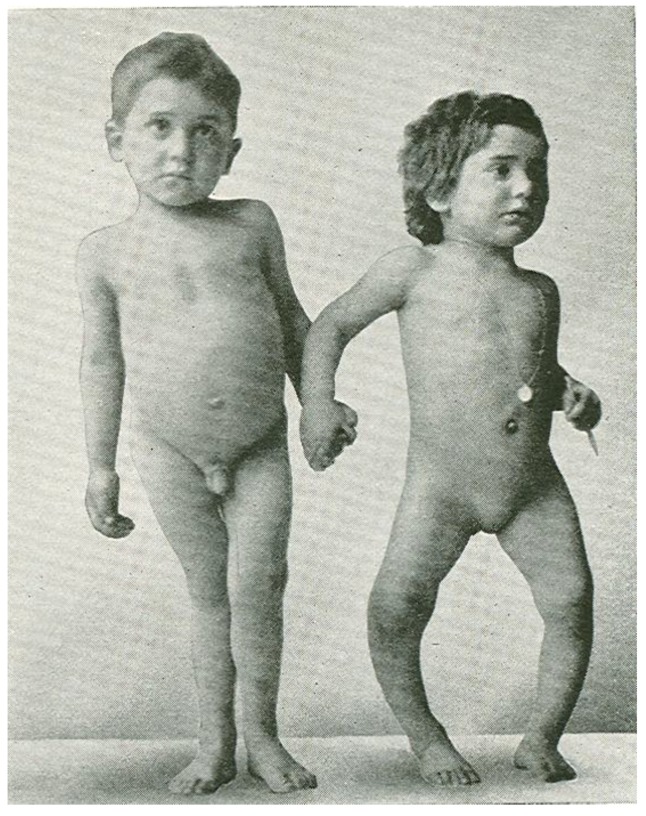
Sister (right) and brother (left) ages 4 years and 6.5 years, respectively, demonstrating classic knock-knees and bow legs, growth retardation, and other skeletal deformities [14]. Note: This figure is reproduced with permission from [14], Copyright © 2006 American Society for Clinical Investigation.

In the mid-1600s most children living in the crowded and polluted industrialized cities in Northern Europe developed a severe bone-deforming disease, rickets, that was characterized by growth retardation, enlargement of the epiphyses of the long bones, deformities of the legs, bending of the spine, knobby projections of the ribcage, and weak and toneless muscles [14,76] (Figure 4). Autopsy studies in children in the Netherlands and Boston in the early 1900s showed a rickets prevalence of 80%–90% [14]. In the 19th and 20th century, the major discoveries regarding the pathogenesis and prevention of rickets were made. In 1822, the importance of sun exposure for the prevention and cure of rickets was recognized by Sniadecki [77]. In 1890, these observations were extended and the recommendation of sun baths to prevent rickets was promoted by Palm [78]. In 1919, Huldschinski [79,80] found that exposing children to UV radiation from a sun quartz lamp (mercury arc lamp) or carbon arc lamp was effective in treating rickets. In 1918, Mellanby *et al.* [81] prevented rickets in puppies with cod liver oil. McCollum *et al.* [82] called this new nutritional factor vitamin D. Hess and Weinstock [83] and Steenbock and Black [84] observed that UV irradiation of various foods and oils imparted antirachitic activity [14].

Vitamin D sufficiency is pivotal for normal skeletal development both *in utero* [7,85] and in childhood [14], and for achieving and maintaining bone health in adults [23]. This is due to the fact that vitamin D sufficiency leads to an adequate calcium-phosphorus product (Ca^2+^ × HPO4^2−^) resulting in an effective bone mineralization [14]. Maternal vitamin D insufficiency during pregnancy was associated with a significant reduction in bone mineral acquisition in infants [85] that still persisted 9 years after birth [86]. In children whose epiphyseal plates haven’t closed, vitamin D deficiency with 25(OH)D levels <15 ng/mL causes chondrocyte disorganization and hypertrophy at the mineralization front as well as skeletal mineralization defects. This results in bone deformities and short stature, the typical signs of vitamin D deficiency rickets [14,87].

In adults low 25(OH)D and high PTH also lead to a low serum calcium × phosphorus product, resulting in osteomalacia, *i.e.*, a defective mineralization of the collagen matrix causing a reduction of structural support and being associated with an increased risk of fracture [17,28]. Results from the National Health and Nutrition Examination Survey III (NHANES III) showed that bone density in the hip was directly related to the serum 25(OH)D level in both genders of all ethnicities [88,89]. A German study examined 25(OH)D serum levels and transiliac crest bone specimens of 675 individuals mainly in the 6th and 7th decade of life (401 males, mean age 58.7 ± 17 years, and 274 females, mean age: 68.3 ± 17.3 years) dying of unnatural death, such as a motor vehicle accident. The bone biopsies were taken within 48 h after death as well as the blood samples. Various previous experiments had shown that the 25(OH)D serum levels were stable for at least 10 days postmortem. While there’s no uniformly accepted osteoid volume cut-off for the histologic diagnosis of osteomalacia, the study showed a prevalence of osteomalacia of over 25% when using a threshold of >2% osteoid volume/bone volume (OV/BV) for the diagnosis of osteomalacia and a prevalence of >43% when using a threshold of 1.2% OV/BV as described by Delling in 1975 [90]. Osteomalacia was absent in all individuals with 25(OH)D >30 ng/mL, suggesting this as minimum serum level for maintenance of bone health. However, no minimum 25(OH)D level could be determined that was inevitably associated with mineralization defects [91].

One possible explanation is that obtaining a single blood level of 25(OH)D doesn’t provide information about the long-term vitamin D status of the individual. It is possible that for example that the subject became ill during the winter and stopped ingesting foods containing vitamin D or decreased sun exposure during the summer that would acutely lower blood levels of 25(OH)D without causing osteomalacia.

## 8. Osteoporosis and Fractures

As a decrease in 25(OH)D leads to secondary hyperparathyroidism associated with osteoclastogenesis and an increase in bone resorption exceeding osteoblast-mediated bone formation [88], this can precipitate and exacerbate osteopenia and osteoporosis in adults [17,92,93].

Osteoporosis has a prevalence of ~1/3 in women 60–70 years of age and of ~2/3 in women 80 years of age or older [7]. It’s estimated that currently 10 million Americans have osteoporosis with 1.5 to 2 million osteoporosis-related fractures annually [94]. An osteoporosis-related fracture will be experienced by one in eight men over age 50 years in their lifetime [95].

Vitamin D promotes bone health by maintaining the PTH levels in a physiologically healthy level, stimulating osteoblastic activity, and promoting bone mineralization as well as reducing risk of falls thereby reducing risk of fracture [93,96].

According to data from the Women’s Health Initiative [97], the odds ratio of risk for hip fracture was inversely related to the serum 25(OH)D level [88]. There’s evidence that patients with 25(OH)D levels >30 ng/mL have a lower risk of fracture. Several studies have been conducted to evaluate the effect of vitamin D supplementation on the fracture risk, with some studies showing a significant reduction of the risk of fractures while others didn’t [98]. One of these showed that the supplementation with calcium (1200 mg) and vitamin D_3_ (800 IU/day) decreased the number of hip fractures by 43% (*p* = 0.043) and the total number of nonvertebral fractures by 32% [99]. The RECORD study however, did not show a reduction in fracture risk with supplementation with vitamin D (800 IU/day), or calcium (1000 mg/day), or both [100], but often compliance was poor and serum 25(OH)D levels were not measured at the end of the study in most participants [7,98,100]. A meta-analysis of more than 30,000 participants did show that supplementation with vitamin D (≥792 IU/day) led to a significant reduction in the risk of fracture; the risk of hip fracture was reduced by 30%, the risk of any non-vertebral fracture by 14% [98,99,100,101,102,103,104,105,106].

## 9. Muscular Health and Falls

Vitamin D exerts multiple effects on muscle health [107]. Its active form 1,25(OH)_2_D could be produced locally in muscle cells as suggested by the recent identification of CYP27B1 bioactivity in regenerating mouse muscle and skeletal muscle cells [108], however other studies have failed to detect this enzyme in muscle cells [109]. 1,25(OH)_2_D is thought to modulate muscle function via the VDR, which seems to be expressed in skeletal muscles [109,110,111,112,113], by regulating gene transcription and promoting *de-novo* protein synthesis [107]. Also, rapid non-genomic pathways involving a membrane-bound vitamin D receptor could exist and affect the calcium handling involving the sarcoplasmic reticulum and the calcium signaling in muscle cells [109]. Several studies indicate that the muscle function depends on the VDR genotype in the muscle cell [114,115]. The possibility of a direct interaction between 25(OH)D and the VDR has been proposed in CYP27B1^−/−^ cells [109,116]. However, the existence of a VDR in muscle cells is discussed highly controversially, as a more recent study failed to detect the VDR in muscle cells and as the antibodies used for immunocytochemical staining to detect the VDR in previous studies have been shown to be not exclusively specific for the VDR and could explain potentially false-positive results in these previous studies [117].

Vitamin D deficiency is associated with diffuse muscle pain, muscle weakness [7,118], predominantly in the proximal muscle groups [115], and a reduction in performance speed [107,119]. This is caused by muscle atrophy of mainly type II muscle fibers [115]. Proximal muscle weakness in severe vitamin D deficiency could also be caused by secondary hyperparathyroidism and resultant hypophosphatemia [60,106,120].

There is a positive association between 25(OH)D, lower extremity function, proximal muscle strength and physical performance [107,121,122]. Muscle strength [123] and postural and dynamic balance [124] were increased by vitamin D supplementation [107]. The effect of vitamin D supplementation on the risk of falls was examined in a randomized, controlled multi-dose study, showing that the supplementation of 800 IU/day lowered the adjusted-incidence rate ratio of falls by 72% compared to those taking placebo over 5 months [125]. A meta-analysis of 8 randomized controlled trials (*n* = 2426) showed that supplemental vitamin D of 700–1000 IU/day or a serum 25(OH)D of ≥24 ng/mL reduced the risk of falls by 19% and 23% respectively. No benefit was observed with lower supplemental doses or lower serum 25(OH)D concentrations [126].

## 10. Cancer

Living at higher latitudes with lower UV exposure and thus lower vitamin D production is associated with an increased risk for the occurrence of a variety of cancers and with an increased likelihood of dying from them, as compared to living at lower latitudes [7,17,127,128]. A recent review of ecological studies associating solar UVB exposure-vitamin D and cancers found strong inverse correlations with solar UVB irradiance for 15 types of cancer: bladder, breast, cervical, colon, endometrial, esophageal, gastric, lung, ovarian, pancreatic, rectal, renal, and vulvar cancer; and Hodgkin’s and non-Hodgkin’s lymphoma [129].

An inverse association between 25(OH)D and the incidence of several cancers and mortality from these cancers has been shown in case-control studies, prospective and retrospective studies [130,131,132,133,134,135,136,137,138,139,140], especially for cancers of the colon, breast and prostate [7]. Regarding colon cancer, the Nurses’ Health cohort study (*n* = 32,826) showed an inverse association of the odds ratios for colorectal cancer with the median 25(OH)D serum levels. At 16.2 ng/mL the odds ratio was 1 and 0.53 at 39.9 ng/mL (*p* ≤ 0.01) [7,140].

These associational studies have certain limitations regarding the establishment of a causality between vitamin D status and a reduced risk of cancer, e.g., as low serum 25(OH)D levels are also linked with confounding factors related to higher cancer risk, including obesity (vitamin D is sequestered in adipose tissue), and lack of physical activity (correlated with less time outdoors and less solar exposure) [138]. However, a population-based, double-blind, randomized placebo-controlled trial of 4 years duration with more than thousand postmenopausal women, whose principal secondary outcome was cancer incidence, showed that the supplementation with calcium (1400–1500 mg/day) and vitamin D_3_ (1100 IU/day) reduced the relative risk (RR) of cancer by ~60% (*p* < 0.01). The repetition of a cancer free survival analysis after the first 12 months revealed, that the relative risk for the calcium + vitamin D group was reduced by ~77% (confidence interval [CI]: 0.09–0.60; *p* < 0.005). Multiple regression models also showed that both treatment and serum 25(OH)D concentrations were significant, independent predictors of cancer risk [137].

**Figure 5:**
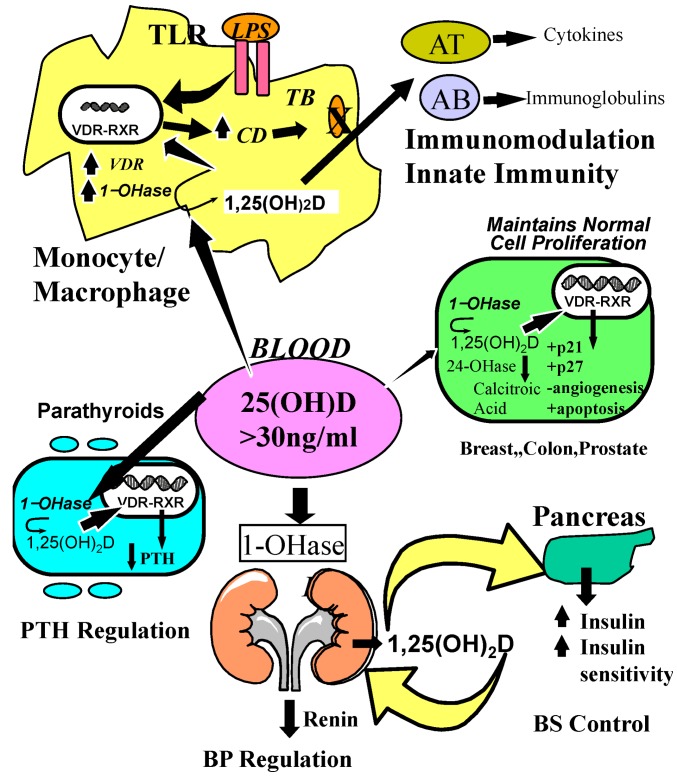
Metabolism of 25-hydroxyvitamin D [25(OH)D] to 1,25 dihydroxyvitamin D 1,25(OH)_2_D for non-skeletal functions. When a monocyte/macrophage is stimulated through its toll-like receptor 2/1 (TLR2/1) by an infective agent such as Mycobacterium tuberculosis (TB), or its lipopolysaccharide (LPS) the signal upregulates the expression of vitamin D receptor (VDR) and the 25-hydroxyvitamin D-1-hydroxylase (1-OHase). 25(OH)D levels >30 ng/mL provides adequate substrate for the 1-OHase to convert it to 1,25(OH)_2_D. 1,25(OH)_2_D returns to the nucleus where it increases the expression of cathelicidin which is a peptide capable of promoting innate immunity and inducing the destruction of infective agents such as TB. It is also likely that the 1,25(OH)_2_D produced in the monocytes/macrophage is released to act locally on activated T (AT) and activated B (AB) lymphocytes which regulate cytokine and immunoglobulin synthesis respectively [143,144,145,146,147]. When 25(OH)D levels are ~30 ng/mL, it reduces risk of many common cancers [130,131,132,133,134,135,136,137,138,139,140]. It is believed that the local production of 1,25(OH)_2_D in the breast, colon, prostate, and other cells regulates a variety of genes that control proliferation. Once 1,25(OH)_2_D completes the task of maintaining normal cellular proliferation and differentiation, it induces the 25-hydroxyvitamin D-24-hydroxylase (24-OHase). The 24-OHase enhances the metabolism of 1,25(OH)_2_D to calcitroic acid which is biologically inert [7,18]. Thus, the local production of 1,25(OH)_2_D does not enter the circulation and has no influence on calcium metabolism. The parathyroid glands have 1-OHase activity [45] and the local production of 1,25(OH)_2_D inhibits the expression and synthesis of PTH [74]. The production of 1,25(OH)_2_D in the kidney enters the circulation and is able to downregulate renin production in the kidney [148,149] and to stimulate insulin secretion in the β-islet cells of the pancreas [148,150]. Note: This figure is reproduced with permission from [21], Copyright © 2007 Michael F. Holick.

Mounting evidence suggests a biological plausibility for anti-carcinogenic effects of vitamin D, which could explain these results. 1,25(OH)_2_D, which has been shown to be produced locally by various cancer cells metabolizing the substrate 25(OH)D [38], inhibits carcinogenesis by several mechanisms [141]. 1,25(OH)_2_D exerts anti-proliferative effects on cancer cells by promoting cyclin-dependent kinase (CDK) inhibitor synthesis, and by influencing several growth factors and their signaling pathways including insulin-like growth factor 1 (IGF-1), transforming growth factor β (TGFβ), Wnt/β-catenin, MAP kinase 5 (MAPK5) and nuclear factor κB (NF-kB) [142] (Figure 5).

Apoptosis is characterized as programmed cell death permitting the removal of damaged cells including cancer cells in multicellular organisms without impairing the cellular microenvironment. Defective apoptosis plays a major role in the development and progression of cancer [151]. It has been shown, that both immunobiological mechanisms of cancer immunosurveillance and cancer immunoediting [152], as well as chemotherapeutic agents and radiation, utilize the apoptotic pathway to induce cancer cell death [151,153]. 1,25(OH)_2_D_3_ might exert anti-carcinogenic effects by promoting various pro-apoptotic mechanisms including the downregulation of the anti-apoptotic gene Bcl-2 [154] and by upregulating of the pro-apoptotic gene Bax [155], 1,25(OH)_2_D_3_ induces differentiation, partly by reducing the expression of the *c-myc* oncogene [141,156]. It regulates the prostaglandin (PG) metabolism and signaling, thus decreasing PG-mediated promotion of carcinogenesis [141,157]. It suppresses tumor angiogenesis, e.g., mediated by 1,25(OH)_2_D’s effects on the PG synthesis and by regulating the expression of crucial factors controlling the angiogenesis. 1,25(OH)_2_D_3_ suppresses tumor invasion and metastasis by various mechanisms [141], e.g., by decreasing the expression and activity of cell invasion-associated serine proteases and metalloproteinases and inducing their inhibitors [158], and by inducing *E*-cadherin expression, contributing to adhesive properties of cells [141,159]. Other effects mediated by 1,25(OH)_2_D are thought to be the induction of autophagy as process to trigger the death of cancer cells and to block tumor growth and by inducing enzymes involved in antioxidant defense mechanisms and DNA-repair [142]. 1,25(OH)_2_D also regulates androgen and estrogen receptor signaling, thereby inhibiting tumor growth of some sex hormone-dependent tumors such as prostate and breast cancer. It has also been shown to reduce the expression of aromatase, thereby inhibiting breast cancer growth [141].

## 11. Vitamin D and Cardiovascular Risk

Most epidemiological and prospective studies as well as meta-analyses [148,160,161,162,163] suggest a significant inverse association between 25(OH)D serum levels and cardiovascular risk. The prospective Intermountain Heart Collaborative Study with more than 40,000 participants revealed that 25(OH)D <15 ng/mL compared to 25(OH)D >30 ng/mL was associated with highly significant increases in the prevalence of type 2 diabetes mellitus, hypertension, hyperlipidemia, and peripheral vascular disease, coronary artery disease, myocardial infarction, heart failure, and stroke (*p* < 0.0001), as well as with incident death (all-cause mortality was used as primary survival measure), heart failure, coronary artery disease/myocardial infarction (*p* < 0.0001), stroke (*p* = 0.003), and their composite (*p* < 0.0001) [164].

A meta-analysis examining the association between vitamin D status and the risk of cerebrovascular events including >1200 stroke cases found that the pooled relative risk for stroke was 52% higher when comparing 25(OH)D levels ≤12.4 ng/mL with 25(OH)D levels >18.8 ng/mL [165].

Many of these associations are well established, causation however is yet to be proven [166]. Individuals spending less time exercising outdoors in the sun, e.g., have a higher risk of developing cardiovascular diseases, and those individuals also will likely have lower 25(OH)D levels coincidentally [166,167]. Also, obesity, a condition associated with cardiovascular disease [168], is associated with a lower vitamin D status due to a sequestration and volumetric dilution of the lipophilic vitamin D in the fat tissue [23,166,169,170], potentially explaining the described correlations [166]. Despite these limitations many studies suggest a biological plausibility for the beneficial effects of vitamin D on cardiovascular risk factors and cardiovascular health.

The vitamin D receptor is present in endothelium, vascular smooth muscle, and cardiomyocytes [162,166] and may protect against atherosclerosis through the inhibition of macrophage cholesterol uptake and foam cell formation, reduced vascular smooth muscle cell proliferation, and reduced expression of adhesion molecules in endothelial cells [166] and through inhibition of cytokine release from lymphocytes [162]. Several meta-analyses indicate an inverse association between vitamin D status and hypertension [171]. Studies showed, that antihypertensive effects were associated with raising 25(OH)D levels with vitamin D supplementation [172,173,174] or UVB exposure [175].

Mechanistically, this effect could be partly mediated by vitamin D’s capability to suppress the levels of PTH, which can cause arrhythmias and lead to myocardial hypertrophy and increased blood pressure [148,176]. 1,25(OH)_2_D_3_ has also been shown to suppress the levels of renin and could contribute to vitamin D’s potential antihypertensive properties [148,149]. 

A meta-analysis examining the association between vitamin D status or vitamin D supplementation, and incident type 2 diabetes showed that individuals with 25(OH)D levels >25 ng/mL compared to those with 25(OH)D <14 ng/mL had a 43% lower risk of developing type 2 diabetes and that a vitamin D supplementation with >500 IU/day compared to <200 IU/day reduced the risk by 13% [177]. In the Nurses’ Health Study >83,000 women were followed-up prospectively and it was shown, that a combined daily intake of >1200 mg calcium and >800 IU vitamin D was associated with a 33% lower risk of type 2 diabetes with RR of 0.67 (CI: 0.49–0.90) compared with an intake of <600 mg calcium and 400 IU vitamin D [178]. A prospective study following-up more than 2000 participants showed, that the risk of progression from prediabetes to diabetes was reduced by 62% when comparing the highest quartile of 25(OH)D levels with the lowest quartile [179,180].

This could be explained by experimental findings indicating that vitamin D exerts various antidiabetic effects. The VDR is expressed in pancreatic beta cells and 1,25(OH)_2_D stimulates insulin secretion [148,150]. Improvement in vitamin D status also leads to a improvement of insulin sensitivity, mediated for example by upregulation of insulin receptors [148], and modulates inflammation, which is also thought to play a role in type 2 diabetes [150,179] (Figure 5).

## 12. Vitamin D’s Role in Autoimmune Disease

Ecological studies have shown that the prevalence of certain autoimmune diseases was associated with latitude, suggesting a potential role of sunlight exposure, and thus vitamin D production, on the pathogenesis of type 1 diabetes mellitus, multiple sclerosis and Crohn’s disease [181]. The increased prevalence at higher latitudes has been shown for multiple sclerosis (MS) [181,182], inflammatory bowel disease [183], rheumatoid arthritis [184] and type 1 diabetes [181,182,185].

A few case-control studies relate the vitamin D status to the risk of developing these autoimmune diseases [181]. One of them, a prospective, nested case-control study analyzed serum samples and the data of disability databases of more than seven million US military personnel, and showed, that among whites (148 cases, 296 controls), the risk of multiple sclerosis significantly decreased with increasing levels of 25(OH)D (odds ratio for a 20 ng/mL increase in 25(OH)D was 0.59 (95% CI: 0.36–0.97). When comparing the highest quintile of 25(OH)D with the lowest, the odds ratio for developing MS was 0.38 (95% CI: 0.19–0.75; *p* = 0.006), with an particularly strong inverse association for 25(OH)D levels measured before age 20 years [186].

A study addressing vitamin D’s effect on multiple sclerosis showed the safety of high-dose vitamin D (~14,000 IU/day). It appeared to have immunomodulatory effects including a persistent reduction in T-cell proliferation and resulted in a trend for fewer relapse events [187]. When examining the association between 25(OH)D serum levels and the relapse rate in MS patients before and after supplementation with ~3000 IU vitamin D per day, a significant strong inverse relationship between the relapse incidence rate and the 25(OH)D level (*p* < 0.0001) was found [188].

An inverse association between maternal 25(OH)D levels and the risk for type 1 diabetes in the offspring has been shown in a population-based, nested cohort study of ~30,000 pregnant women. Compared to the upper quartile of 25(OH)D levels, the odds of type 1 diabetes in the women with the lowest quartile was more than twofold higher [189]. A birth-cohort study with >10,000 children showed, that regular supplementation with 2000 IU vitamin D per day in the first year of life was associated with a 88% reduction of the risk for type 1 diabetes later in life when compared to those without supplementation [190]. However, another study did not show a statistically significant association between taking cod liver oil or other vitamin D supplements in the first year of life and the risk of type 1 diabetes mellitus [191].

Merlino *et al.* [192] showed in a prospective cohort study of 29,368 women of ages 55–69 years without a history of rheumatoid arthritis at study baseline, that greater intake (highest versus lowest tertile) of vitamin D was inversely associated with risk of rheumatoid arthritis (RR 0.67; 95% CI: 0.44–1.00; *p* for trend =0.05).

These associations indicate a contributory role of vitamin D in the pathophysiology of autoimmune diseases. This is further supported by various experimental findings showing vitamin D’s capability to regulate chemokine production, counteracting autoimmune inflammation and to induce differentiation of immune cells in a way that promotes self-tolerance. This involves the enhancement of the innate and the inhibition of the adaptive immune system by regulating the interactions between lymphocytes and antigen presenting cells. By increasing the quantity of Th2 lymphocytes and by inducing proliferation of dendritic cells with tolerance properties, vitamin D exerts anti-inflammatory and immunoregulatory effects [181].

Immune cells possess both the enzymatic machinery to produce 1,25(OH)_2_D and a VDR. This could explain, why certain polymorphisms in the VDR gene seem to affect the risk for multiple autoimmune diseases, the time of onset of disease and disease activity [181,193,194,195,196,197].

## 13. Vitamin D and Infectious Diseases

The plethora of effects of vitamin D on regulating the immune system plays a role in fighting infectious diseases [198]. Vitamin D enhances the innate immunity against various infections [143], especially tuberculosis, influenza and viral upper respiratory tract infections [198].

Historically, cod liver oil (one of only a few natural sources of vitamin D) was given to tuberculosis patients in 19th and 20th century [199,200,201]. Later in the nineteenth century, tuberculosis patients were treated in sanatoriums with heliotherapy, *i.e.*, sun exposure. In 1903, Niels Ryberg Finsen was awarded the Nobel prize for medicine “in recognition of his contribution to the treatment of diseases, especially lupus vulgaris (tuberculosis of the skin), with concentrated light radiation, whereby he has opened a new avenue for medical science” [199,202]. After vitamin D had been identified as the active ingredient in cod-liver oil [199,203], vitamin D_2_ was used successfully in the treatment of lupus vulgaris in several studies. In 1946 a report in *Proc. R. Soc. Med.* [204] stated that there was no room for doubt that calciferol (vitamin D) in adequate dosage will cure a substantial proportion of cases of lupus vulgaris [199,204]. In 1947 the first reference to successful treatment of pulmonary tuberculosis with vitamin D was published [199,205]. In the wake of the antibiotic era both heliotherapy and vitamin D therapy for treating tuberculosis patients were quickly forgotten [199,206]. However recent studies have suggested that vitamin D may have an important role to play in reducing risk for acquiring one of the most common and deadly infectious diseases that plague third world countries [206].

One case-control study examining the association between vitamin D status and tuberculosis showed, that the mean 25(OH)D levels were statistically significant different (*p* < 0.005) between patients with pulmonary and extrapulmonary tuberculosis (10.7 ng/mL) and controls (19.5 ng/mL) [207]. In another study, 25(OH)D levels <10 ng/mL were significantly associated with active tuberculosis (OR 2.9; 95% Cl: 1.3–6.5; *p* = 0.008) [208]. A meta-analysis showed, that low serum 25(OH)D levels were associated with higher risk of active tuberculosis, and that the pooled effect size in random effects meta-analysis was 0.68 (95% CI: 0.43–0.93), representing a medium to large effect [209]. A double-blind, placebo-controlled study in Mongolian school children (*n* = 120) examining the effect of vitamin D supplementation (800 IU/day) on tuberculin skin test conversion to positive showed a trend towards fewer conversions in the vitamin D group (*p* = 0.06), suggesting a potential role of vitamin D in reducing the rate of acquisition of latent tuberculosis infection [210].

Several interventional studies examining the effect of vitamin D supplementation in patients with active tuberculosis have been conducted. Some of them showed an improved immunity against mycobacteria [211], a significantly improved sputum conversion rate and a higher rate of radiological improvement [212], and a significantly hastened sputum culture conversion in participants with the tt genotype of the TaqI vitamin D receptor polymorphism [213]. There was also a higher rate of tuberculosis symptom improvement and a significantly higher weight gain (*p* < 0.005) in children [214]. A prospective, randomized placebo-controlled trial examining the effect of adjunctive vitamin D supplementation in patients receiving antimicrobial therapy showed that vitamin D supplementation led to an accelerated sputum smear conversion and an accelerated resolution of inflammation [215]. Another study however in which three doses of 100,000 IU vitamin D_3_ each were given during 8 months did not lead to a reduction in the clinical severity score or mortality [216].

Some studies examined the effect of vitamin D supplementation on the risk of influenza [217,218].

In 1981, R. Edgar Hope-Simpson proposed that a “seasonal stimulus” was intimately associated with solar radiation and explained the remarkable seasonality of epidemic influenza [219,220]. As the vitamin D status changes during the seasons, it has been suggested, that vitamin D could be this “seasonal stimulus” [219]. A randomized trial of vitamin D_3_ supplementation (1200 IU/day) in school children (*n* = 334) showed a significantly reduced risk for influence A as determined by both antibody and sputum testing compared to the placebo group (RR 0.58; 95% CI: 0.34–0.99; *p* = 0.04) [218].

One study using questionnaires to retrospectively determine the occurrence of influenza-like disease in participants of 10 different clinical trials (*n* = 569), receiving 1111–6800 IU/day, however did not show a significant difference in the incidence and severity of influenza-like disease [217].

The NHANES III study (*n* > 18) revealed an inverse association between serum 25(OH)D levels and recent upper respiratory tract infections (URTI). Lower 25(OH)D levels were independently associated with recent URTI compared with 25(OH)D levels of ≥30 ng/mL (OR 1.36; 95% CI: 1.01–1.84 for <10 ng/mL and OR 1.24; 95% CI: 1.07–1.43 for 10 to <30 ng/mL). In individuals with asthma or chronic obstructive airway disease this association was stronger (OR of 5.67 in asthma respectively OR of 2.26 in chronic obstructive airway disease) [221]. A study in Finish men (*n* = 800) found a significant association between 25(OH)D serum levels <16 ng/mL and significantly more days of absence from duty due to respiratory infections (*p* = 0.004) [222]. In Indian children (*n* = 150) vitamin D deficiency has been associated with a significantly higher risk of acute lower respiratory infections [223].

A study with >200 participants whose primary endpoint was the effect of vitamin D supplementation on bone loss also revealed, that the vitamin D_3_ supplementation for 2 years with 800 IU/day and for 1 year with 2000 IU/day was associated with a significantly reduced risk of cold and influenza symptoms, an effect that was magnified with the supplementation of 2000 IU/day [198,224]. Other studies however did not show a statistically significant difference, possibly due to poor compliance [225,226]. Certain VDR polymorphisms were also associated with a significantly increased risk of acute lower respiratory tract infections [227].

Several mechanisms could explain vitamin D’s potentially beneficial effects on infectious diseases. Monocytes and macrophages can sense pathogen-associated molecular patterns (PAMPs) of, e.g., tuberculosis by utilizing their toll-like receptors (TLRs). This induces both VDR and CYP27B1, which increases the local production of 1,25(OH)_2_D that is dependent on the serum 25(OH)D concentration [145,228]. 1,25(OH)_2_D enhances the innate immune system by inducing the production of antimicrobial peptides like cathelicidin, reactive oxygen species by the (reduced) nicotinamide adenine dinucleotide phosphate (NADPH) oxidase and potentially reactive nitrogen species by inducible nitric oxide synthase (iNOS), and by inducing autophagy [143,144,145,146,147] (Figure 4).

## 14. Vitamin D and Respiratory Diseases

Although some studies did not find a consistent association between 25(OH)D levels in cord blood, maternal vitamin D intake or status during pregnancy and the risk for asthma in childhood [229,230,231,232,233,234,235,236], in children with asthma, 25(OH)D levels seem to correlate positively with asthma control [237] and lung function [238], and inversely with corticosteroid use [239]. A few interventional studies examining vitamin D’s effect on asthma exist [229]. One of them showed as secondary outcome that vitamin D_3_ supplementation (1200 IU/day) in school children was associated with a significant 83% reduced risk for asthma exacerbations [218]. Presumably vitamin D’s immunmodulatory and pulmonary effects could play a role [229].

## 15. Prevention and Treatment of Vitamin D Deficiency

**Figure 6:**
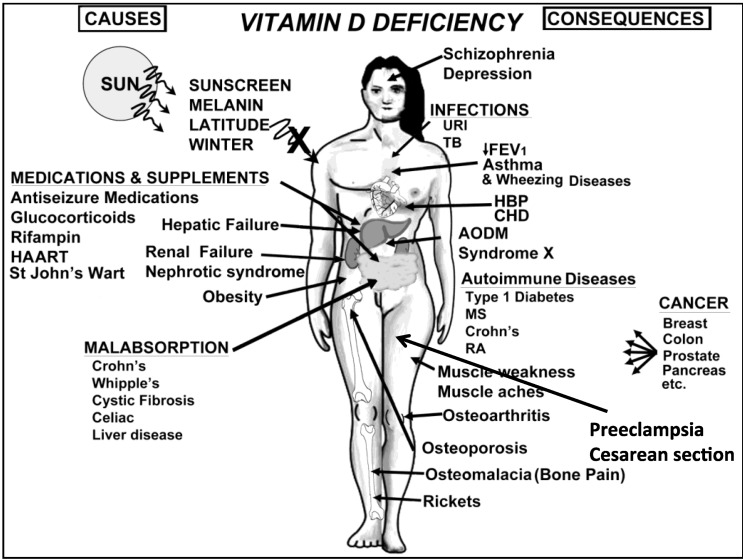
A Schematic representation of the major causes for vitamin D deficiency and potential health consequences. Note: This figure is reproduced with permission from [21], Copyright © 2007 Michael F. Holick.

According to the Endocrine Society Practice Guidelines a screening for vitamin D deficiency by measuring the 25(OH)D serum level is only recommended for individuals at risk (the most important risk factors are listed in Figure 6), and not for the general population [16]. To prevent vitamin D deficiency, the Institute of Medicine (IOM) recommends, that infants should immediately receive a daily supplementation of vitamin D of 400 IUs during the first year of life. Individuals between 1 and 70 years should receive 600 IU of vitamin D daily and adults >70 years should receive a daily dose of 800 IU vitamin D [53] (Table 2). The serum 25(OH)D level increases for every 100 IU/day by ~0.6–1.0 ng/mL [29,37,240,241]. The doses recommended by IOM will likely increase the 25(OH)D level to 20 ng/mL, which they considered to be adequate for bone health, but not to levels >30 ng/mL, as recommended by the Endocrine Society.

That’s why the Endocrine Society recommended in its Practice Guidelines that infants during their first year of life receive a daily supplementation of 400–1000 IU (up to 2000 IU is safe), children and adolescents between 1 and 18 years a daily supplementation of 600–1000 IU (up to 4000 IU is safe), and adults >18 years a daily supplementation of 1500–2000 IU (up to 10,000 IU is safe) for the prevention of vitamin D deficiency [16,53] (Table 2).

**Table 2:** Recommendations of the Institute of Medicine and the Endocrine Society Practice Guidelines for daily vitamin D supplementation to prevent vitamin D deficiency. This table is reproduced with permission from [16], Copyright © 2011 The Endocrine Society.

	IOM Recommendations				Endocrine Society’s Recommendations	
Life Stage Group	AI	EAR	RDA	UL	Daily Allowance (IU/day)	UL (IU)
*Infants*						
0 to 6 months	400 IU (10 μg)			1000 IU (25 μg)	400–1000	2000
6 to 12 months	400 IU (10 μg)			1500 IU (38 μg)	400–1000	2000
*Children*						
1–3 years		400 IU (10 μg)	600 IU (15 μg)	2500 IU (63 μg)	600–1000	4000
4–8 years		400 IU (10 μg)	600 IU (15 μg)	3000 IU (75 μg)	600–1000	4000
*Males*						
9–13 years		400 IU (10 μg)	600 IU (15 μg)	4000 IU (100 μg)	600–1000	4000
14–18 years		400 IU (10 μg)	600 IU (15 μg)	4000 IU (100 μg)	600–1000	4000
19–30 years		400 IU (10 μg)	600 IU (15 μg)	4000 IU (100 μg)	1500–2000	10,000
31–50 years		400 IU (10 μg)	600 IU (15 μg)	4000 IU (100 μg)	1500–2000	10,000
51–70 years		400 IU (10 μg)	600 IU (15 μg)	4000 IU (100 μg)	1500–2000	10,000
>70 years		400 IU (10 μg)	800 IU (20 μg)	4000 IU (100 μg)	1500–2000	10,000
*Females*						
9–13 years		400 IU (10 μg)	600 IU (15 μg)	4000 IU (100 μg)	600–1000	4000
14–18 years		400 IU (10 μg)	600 IU (15 μg)	4000 IU (100 μg)	600–1000	4000
19–30 years		400 IU (10 μg)	600 IU (15 μg)	4000 IU (100 μg)	1500–2000	10,000
31–50 years		400 IU (10 μg)	600 IU (15 μg)	4000 IU (100 μg)	1500–2000	10,000
51–70 years		400 IU (10 μg)	600 IU (15 μg)	4000 IU (100 μg)	1500–2000	10,000
>70 years		400 IU (10 μg)	800 IU (20 μg)	4000 IU (100 μg)	1500–2000	10,000
*Pregnancy*						
14–18 years		400 IU (10 μg)	600 IU (15 μg)	4000 IU (100 μg)	600–1000	4000
19–30 years		400 IU (10 μg)	600 IU (15 μg)	4000 IU (100 μg)	1500–2000	10,000
31–50 years		400 IU (10 μg)	600 IU (15 μg)	4000 IU (100 μg)	1500–2000	10,000
*Lactation* *						
14–18 years		400 IU (10 μg)	600 IU (15 μg)	4000 IU (100 μg)	600–1000	4000
19–30 years		400 IU (10 μg)	600 IU (15 μg)	4000 IU (100 μg)	1500–2000	10,000
31–50 years		400 IU (10 μg)	600 IU (15 μg)	4000 IU (100 μg)	1500–2000	10,000

* Mother’s requirement 4000–6000 (mother’s intake for infant’s requirement if infant is not receiving 400 IU/day); AI = Adequate Intake; EAR = Estimated Average Requirement; IU = International Units; RDA = Recommended Dietary Allowance; UL = Tolerable Upper Intake Level.

However, obese individuals, patients with malabsorption syndromes, and patients on glucocorticoids, anti-seizure and AIDS medications may require higher doses of vitamin D than individuals without these conditions [16]. The Endocrine Society’s Clinical Practice Guidelines also recommended sensible sun exposure, which for most individuals is the main physiological source of vitamin D, and provided a list of the foods rich in vitamin D, and encouraged taking a daily vitamin D supplement to ensure adequate 25(OH)D levels.

**Figure 7:**
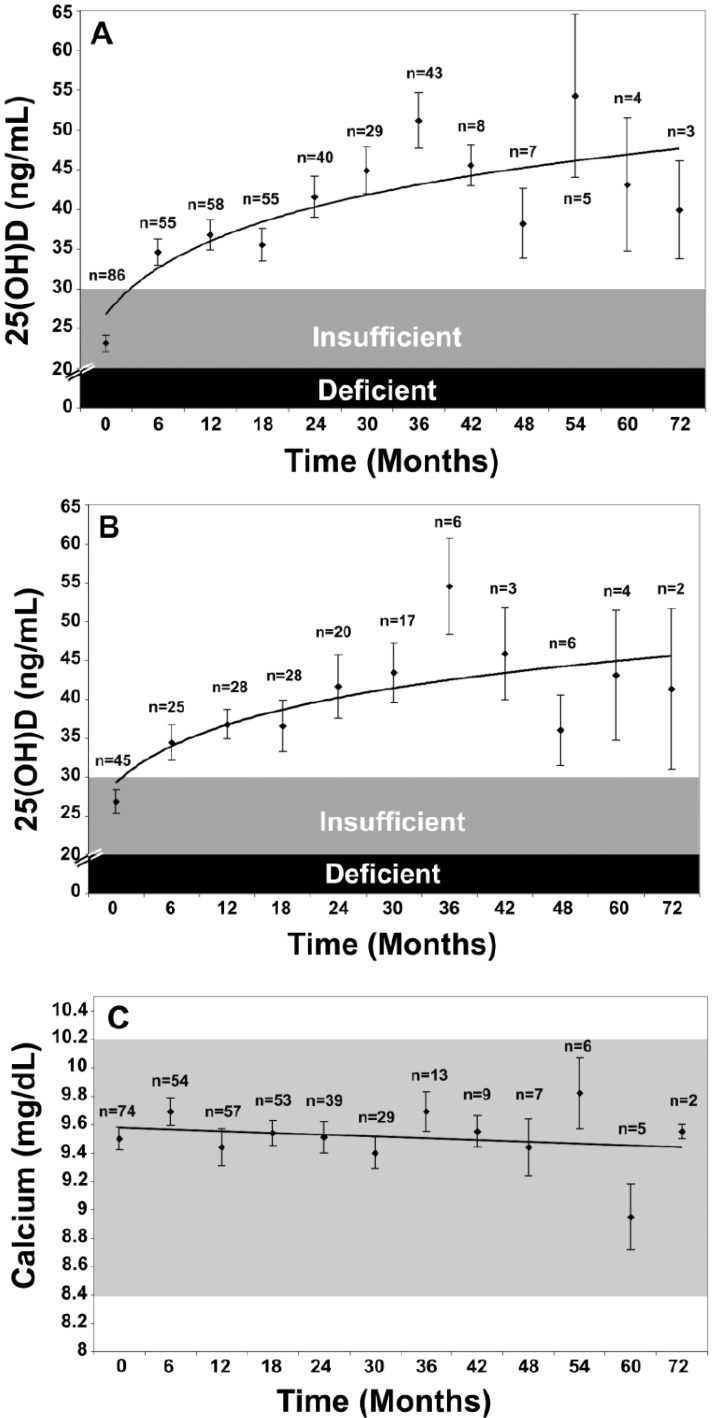
(**A**) Mean serum 25-hydroxyvitamin D [25(OH)D] levels in all patients: includes patients treated with 50,000 IU vitamin D_2_ every 2 weeks (maintenance therapy, *n* = 81), including those patients with vitamin D insufficiency who were initially treated with 8 weeks of 50,000 IU vitamin D_2 _weekly prior to maintenance therapy (*n* = 39). Error bars represent standard error of the mean, mean result over 5 years shown. Time 0 is initiation of treatment, results shown as mean values averaged for 6 month intervals. When mean 25(OH)D in each 6 month group was compared to mean initial 25(OH)D, a significant difference was shown with *p* < 0.001 up until month 43 and *p* < 0.001 when all remaining values after month 43 were compared to mean initial 25(OH)D. (**B**) Mean serum 25(OH)D levels in patients receiving maintenance therapy only: Levels for 37 patients who were vitamin D insufficient (25(OH)D levels <30 ng/mL) and 5 patients who were vitamin D sufficient (25(OH)D levels ≥30 ng/mL) who were treated with maintenance therapy of 50,000 IU vitamin D_2_ every two weeks. Error bars represent standard error of the mean, mean result over 5 years shown. Time 0 is initiation of treatment, results shown as mean values averaged for 6 month intervals. When mean 25(OH)D in each 6 month group were compared to mean initial 25(OH)D, a significant difference was shown with *p* < 0.001 up until month 37 and *p* < 0.001 when all remaining values after month 43 were compared to mean initial 25(OH)D. (**C**) Serum calcium levels: Results for all 81 patients who were treated with 50,000 IU of vitamin D_2_. Error bars represent standard error of the mean. Time 0 is initiation of treatment, results shown as mean values averaged for 6 month intervals. Normal serum calcium: 8.5–10.2 mg/dL.Note: This figure is reproduced with permission from [242], Copyright © 2009 American Medical Association.

The Endocrine Society’s Practice Guidelines also recommended treatment strategies for patients with vitamin D deficiency depending on age and underlying medical conditions. For vitamin D deficient infants 0–1 years old, a treatment with 2000 IU/day of vitamin D_2_ or vitamin D_3_ or with 50,000 IU of vitamin D_2_ or vitamin D_3_ once weekly for 6 weeks was suggested, followed by maintenance therapy of 400–1000 IU/day. For vitamin D deficient children aged 1–18 years who are vitamin D deficient, treatment with 2000 IU/day of vitamin D_2_ or vitamin D_3_ or with 50,000 IU of vitamin D_2_ once a week, both for at least 6 weeks, was suggested, followed by maintenance therapy of 600–1000 IU/day. Vitamin D deficient adults should be treated with 50,000 IU of vitamin D_2_ or vitamin D_3_ once a week for 8 weeks or with ~6000 IU/day of vitamin D_2_ or vitamin D_3_, followed by maintenance therapy of 1500–2000 IU/day. In obese patients, patients with malabsorption syndromes, and patients on medications affecting vitamin D metabolism, two to three times higher doses are (at least 6000–10,000 IU/day) of vitamin D to treat vitamin D deficiency are recommended, followed by maintenance therapy of at least 3000–6000 IU/day [16]. This strategy of giving 50,000 IU of vitamin D twice monthly to treat or prevent recurrence of vitamin D deficiency or insufficiency was without any toxicity for up to six years [242] (Figure 7).

However, certain conditions like granulomatous conditions [243], genetic disorders [244] or rare polymorphisms of enzymes involved in vitamin D metabolism [245] are associated with an increased risk for vitamin D toxicity.

## 16. Conclusion

What continues to be needed are randomized controlled interventional studies with high power and using sufficiently high doses of vitamin D examining vitamin D’s effects on various health outcomes.

However, the present body of evidence of experimental findings, ecological, case-control, retro- and prospective observational and interventional studies is substantial and suggests a pivotal role of vitamin D for a plethora of physiological functions and health outcomes including neuropsychiatric disorders [246], justifying the recommendation to enhance children’s and adults’ vitamin D status by following recommendations for sensible sun exposure, ingesting foods that contain vitamin D and vitamin D supplementation. Increasing the vitamin D status worldwide in the general adult and children population without rare conditions associated with an increased risk for vitamin D toxicity will help improve their overall health and well-being (Figure 6).
